# Clinical efficacy analysis of butylphthalide combined with urinary kallidinogenase in treating chronic cerebral circulatory insufficiency

**DOI:** 10.1002/brb3.2920

**Published:** 2023-02-22

**Authors:** Bo Hu, Feifan Hu, Yulin Qiao

**Affiliations:** ^1^ Department of Neurology Wuhan Asia General Hospital Wuhan China

**Keywords:** butylphthalide, chronic cerebral circulatory insufficiency, curative effect, urinary kallidinogenase

## Abstract

**Objective:**

To investigate the clinical effect of butylphthalide combined with urinary kallidinogenase in the treatment of chronic cerebral circulatory insufficiency (CCCI).

**Methods:**

A total of 102 CCCI patients admitted to our hospital from October 2020 to December 2021 were retrospectively enrolled in this study. According to the different therapeutic strategy, the patients were divided into combined group (treated with butylphthalide combined with urinary kallidinogenase, *n* = 51) and butylphthalide group (treated with butylphthalide, *n* = 51). Blood flow velocity and cerebral blood flow perfusion before and after treatment between the two groups were compared. The clinical efficacy and adverse events of the two groups were analyzed.

**Results:**

After treatment, the effective rate of the combined group was significantly higher than the butylphthalide group (*p* = .015). Before treatment, the blood flow velocity of middle cerebral artery (MCA), vertebral artery (VA), basilar artery (BA) were comparable (*p* > .05, respectively), while after treatment, the blood flow velocity of MCA, VA, and BA in combined group were faster than those in butylphthalide group (*p* < .001, respectively). Before treatment, the relative cerebral blood flow (rCBF), relative cerebral blood volume (rCBV), relative mean transmit time (rMTT) of the two groups were comparable (*p* > .05, respectively). After treatment, rCBF and rCBV in combined group were higher than those in butylphthalide group (*p* < .001, respectively), and rMTT in combined group was lower than that in butylphthalide group (*p* = .001). The rate of adverse events in the two groups were comparable (*p* = .558).

**Conclusion:**

Butylphthalide combined with urinary kallidinogenase can improve the clinical symptoms of CCCI patients, and the effect is promising, which is worthy of clinical application.

## Graphical abstract

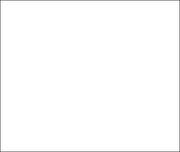

## 1 INTRODUCTION

Chronic cerebral circulatory insufficiency (CCCI) is a common disease in clinical neurology department. It refers to the various causes of cerebrovascular stenosis and (or) low perfusion, reduce the supply of blood flow to the brain tissue, unable to meet the demand of normal brain tissue, on the basis of whole brain blood flow and low metabolism form is given priority to with local pathological changes, causing mild, volatility of brain dysfunction (Chukanova et al., 2021). The main clinical symptoms were memory deterioration, insomnia, vertigo, headache, cognitive decline, etc., which could be confirmed by brain CT and MRI scans, and the patients did not have vascular or organic brain lesions (Ding et al., 2019). However, if it is not treated in time and effectively, it will induce diseases like senile dementia, or cerebral infarction, which will pose a serious threat. Therefore, early detection and treatment can effectively relieve the clinical symptoms of CCCI patients, improve the efficacy, and reduce the incidence of related complications. At present, there is no specific drug treatment for CCCIs in clinical practice, but mainly symptomatic treatment with drugs, including brain protection, improvement of circulation and dilation of blood vessels, etc. Nonpharmacologic treatments include carotid stent implantation, balloon dilation, pulmonary endarterectomy, and intracranial artery bypass grafting to remove the causes of hypoperfusion and improve cerebral circulation (Chernii, 2020; Jia et al., 2020). In recent years, butylphthalide and urinary kallidinogenase treatment for ischemic stroke has been considered as a promising strategy in China. They have the ability to improve cerebral blood flow reserve, promote collateral circulation, protect neurons, and improve the prognosis of patients with ischemic cerebrovascular diseases (Chen et al., 2019; Ni et al., 2021).

Many studies have reported the use of butylphthalide and urinary kallidinogenase in the treatment of cerebral infarction alone or in combination (Wen et al., 2022; Zhai & Yang, 2022), but there are few studies on butylphthalide combined with urinary kallidinogenase in CCCIs. This study aims to investigate the therapeutic effect of butylphthalide combined with urinary kallidinogenase in the clinical treatment of patients with CCCI.

## 2 MATERIALS AND METHODS

### 2.1 General data

From October 2020 to December 2021, a total of 102 CCCI patients were retrospectively enrolled in this study. According to the different therapeutic strategy, the patients were divided into combined group (treated with butylphthalide combined with urinary kallidinogenase, *n* = 51) and butylphthalide group (treated with butylphthalide, *n* = 51). In combined group, there were 27 males and 24 females, with an average age of 61.0 ± 12.4 years (range, 45–76 years). Butylphthalide group includes 26 males and 25 females, with an average age of 60.9 ± 13.3 years (range, 44–75 years).

The study protocol complied with the relevant requirements of the World Medical Association Declaration of Helsinki and was approved by the Ethics Committee of our hospital. Written informed consent was obtained from all patients.

### 2.2 Diagnostic, inclusion, and exclusion criteria

Diagnostic criteria: According to the diagnostic criteria of the Chinese Classification of Cerebrovascular Diseases 2015 (Chinese Society of Neurology, of Chinese Medical Association, Cerebrovascular Group of Chinese Society of Neurology, 2017): (1) patients with head heaviness, dizziness, headache, limb numbness, sleep disorders, mood disorders, inattention, hypomnesis and other chronic brain dysfunction; (2) patients with hypertension, hyperlipidemia, diabetes, and other risk factors for cerebrovascular diseases; (3) patients with no signs of cerebral focal nerve damage; (4) there are a series of evidence supporting cerebral arteriosclerosis such as fundus artery, radial artery, temporal artery, and other peripheral arteriosclerosis changes, or cerebral perfusion artery vascular murmur can be heard, or concomitant cerebral artery stenosis or obstruction detected by cerebral vascular examination; (5) computed tomography angiography (CTA) or (magnetic resonance imaging) MRI showed no vascular organic brain changes, or only slight ischemic changes; (6) cerebral perfusion imaging confirmed the presence of reduced cerebral blood flow or hypoperfusion; (7) the course of the disease was long, and the history was traced back to more than 3 months.

Inclusion criteria: (1) those who meet the above diagnostic criteria; (2) signed the informed consent.

Exclusion criteria: (1) patients with cerebral parasitic diseases, brain trauma, brain tumors, arteriovenous malformations, intracranial aneurysms; (2) patients who cannot accept MRI scan because of the presence of metal implant such as heart stent or steel nail, or the presence of claustrophobic; (3) patients with a history of heart disease such as bradycardia and sick sinus syndrome; (4) patients with creatinine clearance < 30 mL/min; (5) patients with other serious systemic diseases such as blood system; (6) angiotensin converting enzyme inhibitors were used before treatment; (7) patients with severe depression; (8) failure to take medicine as required; (9) patients with serious adverse events.

## 3 METHODS

According to the guidelines in the Chinese Guidelines for Secondary Prevention of Ischemic Stroke and Transient Ischemic Attack (2014 edition) (Chinese Society of Neurology, of Chinese Medical Association, Cerebrovascular Group of Chinese Society of Neurology, 2015), routine treatment including 100 mg aspirin enteric‐coated tablets (manufacturer: Shenyang Ogina Pharmaceutical Co., LTD.; Batch No: 20150903, specification: 100 mg per tablet) oral treatment, once a day; 1.2 g piracetam tablets (Manufacturer: Guangdong Huanan Pharmaceutical Group Co., LTD.; Batch number: 20170822, specification: 0.4 g per tablet) oral treatment, 3 times a day. If the patients complicated with diabetes, hyperlipidemia, or hypertension, then give the corresponding treatment.

Butylphthalide group was given butylphthalide sodium chloride injection on the basis of conventional treatment (manufacturer: Shiyao Group Enbipu Pharmaceutical Co., LTD; Batch No: 6182009138, specification: 100 mL/ bottle), 100 mL/ time, each infusion time > 50 min, twice/day, interval time > 6 h.

Combined group was given urinary kallidinogenase (manufacturer: Guangdong Tempu Biochemical Pharmaceutical Company; Batch number: 3107050331081001, specification: 0.15 PNA unit/bottle) on the basis of the treatment in the control group, and 0.15 PNA unit was added to 100 mL normal saline intravenously for 30 min, once a day. All patients were treated continuously for 14 days.

The use of angiotensin converting enzyme inhibitors (ACEI) was prohibited 24 h before treatment and throughout the medication cycle in all cases.

### 3.1 Observational index

Therapeutic evaluation (Chinese Society of Neurology, of Chinese Medical Association, Cerebrovascular Group of Chinese Society of Neurology, 2015): Obvious effective: after treatment, symptoms such as headache, vertigo, and head heaviness were significantly reduced, the total score of MMSE scale was ≥27, and the CSS score was < 8. Effective: after treatment, symptoms such as headache, vertigo, and head heaviness were significantly reduced, the total score of MMSE scale was 21–26, and the CSS score was 8–15. Ineffective: none of the above symptoms improved or even worsened significantly. Total effective rate = obvious effective + effective rate.

Cognitive function assessment: the MMSE scale total score was used to assess 7 aspects such as immediate memory, temporal orientation, and place orientation, with a total score of 0–30, normal being 27–30, cognitive dysfunction being < 27, and mild cognitive dysfunction being 21–26, the lower the score the more severe the cognitive dysfunction.

Assessment of the degree of neurological deficit: the degree of neurological deficit (CSS) score was used, including walking ability, lower extremity ability, hand muscle strength, upper extremity muscle strength, speech, facial muscle, horizontal gaze function, and consciousness, with a total score of 0–45. The higher the score, the more significant the degree of neurological impairment.

Blood flow velocity: the mean blood flow velocity of left and right middle cerebral arteries (MCA), left and right vertebral arteries (VA), and basilar arteries (BA) was examined by color Doppler ultrasound (Germany DWL, probe frequency 2 MHz).

Evaluation of cerebral blood flow perfusion: before and after treatment, cerebrovascular CT perfusion scan was performed at the level of region of interest (affected side), and the data of the contralateral side (healthy side) were obtained to calculate relative cerebral blood flow (rCBF), relative cerebral blood volume (rCBV) and relative mean transit time (rMTT). *r* value = affected side/ healthy side. rCBF between 0.9 and 1.1 was considered normal perfusion, <0.9 was considered hypoperfusion, and >1.1 was considered hyperperfusion.

Adverse events including liver and kidney injury, rash, arrhythmia, nausea, and vomiting were recorded in the two groups.

### 3.2 Statistical analysis

The enumeration data were expressed as constituent ratio, and the chi‐square test or exact probability method was used to compare the rates between groups. Measurement data conforming to normal distribution were expressed as mean ± standard deviation, and compared using *t* test. Measurement data with skewed distribution were expressed as *M* (Q1–Q3), and the comparison between the two groups was analyzed by Wilcoxon nonparametric test. *p* < .05 was considered significant difference. All statistical analyses were performed using IBM SPSS Statistics 22.0 software (IBM SPSS Statistics).

## 4 RESULTS

### 4.1 Baseline characteristics

There were 51 cases in both groups. The course of disease in combined group was 8.9 ± 1.7 months, while that in butyphthalide group was 8.8 ± 1.8 months. There was no significant difference in gender, age, course of disease, comorbidity, smoking, alcohol drinking, and educational background between the two groups (all *p* > .05). See Table 1.

**TABLE 1:** Comparison of baseline characteristics

	Combined group	Butyphthalide group		
Items	(*n* = 51)	(*n* = 51)	χ^2^/*t*	*p* Value
Gender (male/female)	27/24	26/25	0.039	.843
Age (years)	61.0 ± 12.4	60.9 ± 13.3	0.031	.975
Course of disease (months)	8.9 ± 1.7	8.8 ± 1.8	0.355	.723
Comorbidity (*n*, %)			0.173	.982
*Hypertension*	17 (33.33)	16 (31.37)		
*Diabetes*	14 (27.45)	12 (23.53)		
*Coronary heart disease*	16 (31.37)	15 (29.41)		
*Hyperlipidaemia*	17 (33.33)	18 (35.29)		
Statins use (*n*, %)			0.065	.799
*Atorvastatin*	6 (11.76)	7 (13.73)		
*Rosuvastatin*	4 (7.84)	2 (3.92)		
Smoking (*n*, %)	15 (29.41)	14 (27.45)	0.048	.826
Alcohol drinking (*n*, %)	13 (25.49)	12 (23.53)	0.053	.818
Degree of education (*n*, %)			0.205	.903
*Primary school or below*	12 (23.53)	12 (23.53)		
*Junior high school*	19 (37.25)	21 (41.18)		
*Senior high school or above*	20 (39.22)	18 (35.29)		

### 4.2 Comparison of clinical efficacy

The numbers of obvious effective, effective, ineffective in combined group were 34, 16, and 1, respectively. And the numbers of above indicators in butyphthalide group were 19, 23, and 7, respectively. The total effective rate were 98.04% vs. 86.27% (*p* = .015). Total effective rate in combined group was higher than that in butyphthalide group. See Table 2.

**TABLE 2:** Comparison of clinical efficacy

Clinical efficacy	Combined group (*n* = 51)	Butyphthalide group (*n* = 51)	*Z* value	*p* Value
Obvious effective (*n*)	34	19		
Effective (*n*)	16	23		
Ineffective (*n*)	1	7		
Total effective rate (*n*,%)	50 (98.04)	44 (86.27)	5.971	.015

### 4.3 Comparison of blood flow velocity

Before treatment, the blood flow velocity of MCA (47.98 ± 8.21 vs. 48.02 ± 8.19 cm/s, *p* = .978), VA (23.02 ± 4.39 vs. 22.98 ± 4.41 cm/s, *p* = .963), BA (25.89 ± 4.87 vs. 26.01 ± 4.36 cm/s, *p* = .896) were comparable in the two groups. After treatment, the blood flow velocity of MCA (69.87 ± 8.76 vs. 55.39 ± 8.08 cm/s, *p* < .001), VA (34.28 ± 4.52 vs. 29.76 ± 4.34 cm/s, *p* < .001), BA (40.76 ± 5.08 vs. 33.28 ± 5.03 cm/s, *p* < .001) in combined group were significantly higher than those in butyphthalide group. See Table 3.

**TABLE 3:** Comparison of blood flow velocity

Items	Time point	Combined group (*n* = 51)	Butyphthalide group (*n* = 51)	*t* Value	*p* Value
MCA (cm/s)	Before treatment	47.98 ± 8.21	48.02 ± 8.19	–0.025	.978
	After treatment	69.87 ± 8.76	55.39 ± 8.08	8.677	<.001
VA (cm/s)	Before treatment	23.02 ± 4.39	22.98 ± 4.41	0.046	.963
	After treatment	34.28 ± 4.52	29.76 ± 4.34	5.151	<.001
BA (cm/s)	Before treatment	25.89 ± 4.87	26.01 ± 4.36	–0.131	.896
	After treatment	40.76 ± 5.08	33.28 ± 5.03	7.479	<.001

MCA, middle cerebral artery; VA, vertebral artery; BA, basilar artery.

### 4.4 Comparison of cerebral blood perfusion

Before treatment, the rCBF (0.56 ± 0.13 vs. 0.57 ± 0.14, *p* = .709), rCBV (0.71 ± 0.14 vs. 0.72 ± 0.16, *p* = .738), rMTT (1.61 ± 0.36 vs. 1.59 ± 0.34, *p* = .774) of the two groups were comparable. After treatment, the rCBF (0.79 ± 0.11 vs. 0.66 ± 0.12, *p* < .001), rCBV (0.96 ± 0.13 vs. 0.83 ± 0.15, *p* < .001) in combined group were significantly high than those in butyphthalide group. rMTT (1.13 ± 0.23 vs. 1.29 ± 0.26, *p* = .001) in combined group was significantly lower than that in butyphthalide group. See Table 4.

**TABLE 4:** Comparison of cerebral blood perfusion

Items	Time point	Combined group (*n* = 51)	Butyphthalide group (*n* = 51)	*t* Value	*p* Value
rCBF	Before treatment	0.56 ± 0.13	0.57 ± 0.14	–0.374	.709
	After treatment	0.79 ± 0.11	0.66 ± 0.12	5.703	<.001
rCBV	Before treatment	0.71 ± 0.14	0.72 ± 0.16	–0.336	.738
	After treatment	0.96 ± 0.13	0.83 ± 0.15	4.677	<.001
rMTT	Before treatment	1.61 ± 0.36	1.59 ± 0.34	0.288	.774
	After treatment	1.13 ± 0.23	1.29 ± 0.26	–3.292	.001

rCBF, relative cerebral blood flow; rCBV, relative cerebral blood volume; rMTT, relative mean transmit time.

### 4.5 Adverse events

One case (1/51, 1.96%) in combined group suffered facial fever during the treatment, and the symptom lasted for about 10 min and then relieved, without stopping the drug or giving other treatments. In butyphthalide group, there was 1 case of nausea, 1 patient's heart rate increased and blood pressure decreased. After adjusting fluid velocity, the patient's symptoms improved and blood pressure increased, no recurrence of discomfort later. The incidence of adverse events in butyphthalide group was 3.92% (2/51). There was no significant difference of adverse events incidence between the two groups (χ^2^ = 0.343, *p* = .558).

## 5 DISCUSSION

CCCI is a common disease in neurology. Its etiology is related to blood pressure level, arterial thrombosis, vascular function, blood flow velocity, blood viscosity, cardiac output (Hynes et al., 2019). According to the data of the World Health Organization, CCCI patients have a significantly higher risk of progressing to serious cardiovascular events within 1–5 years than normal people, and can easily induce cognitive impairment and even dementia. Therefore, CCCI is also considered as a pre‐stroke state (Yu et al., 2022). CCCI has certain reversibility. Timely administration of effective treatment strategy and drugs can effectively prevent progressive stroke or dementia, and play a positive role in improving the prognosis of patients. Butylphthalide is a new class I drug launched in China in recent years, which can help patients improve brain energy metabolism and microcirculation (Jia et al., 2020). Butylphthalide has the following pharmacological mechanisms (Ding et al., 2020): ① Increase ischemic blood flow and improve microcirculation in ischemic position. ② Protect the neurons in the ischemic position and improve the function of nerve defects. ③ Improve the activity of antioxidant enzymes, assist free radical scavenging, and reduce oxidative stress damage. ④ Reduce edema in ischemic position and promote edema subsidence in infarcted position. Butylphthalide has high selectivity for blood vessels, which can improve the endothelial function of micro‐vessels, restore the diameter of collateral circulation tube, and improve the blood flow velocity in the infarcted area, so as to promote the regeneration of collateral vessels. In addition, butylphthalide has no obvious effect on blood pressure, drug interaction is minor, and has high safety. Yuan (2020) pointed out that the application of butylphthalide in the treatment of CCCI can improve the control of inflammation, improve cerebral blood flow, and improve the treatment effect, without increasing adverse events. Cheng et al. (2019) indicated that butylphthalide can effectively reduce the blood lipid level and improve cerebral blood flow, with definite clinical effect, which is worthy of application and promotion in the treatment of CCCI.

Urinary kallidinogenase can be converted into kinin and vasodilator in vivo, selectively dilating the arterioles of ischemic brain tissue, and increasing the blood flow to the brain and the level of hemoglobin in the cerebral blood, thereby improving the blood supply and oxygen supply of the ischemic brain tissue. Also, it can upregulate the expression of vascular endothelial growth factor, promote vascular endothelial growth factor, promote angiogenesis, increase cerebral blood flow reserve, and improve the effect of hypoperfusion (Ni et al., 2021). So far, research on urinary kallidinogenase mainly focuses on cerebral infarction (Dong et al., 2020; Ni et al., 2021), but few studies on CCCI are available. The results of this study showed that after treatment, the total effective rate of the combined group was significantly higher than that of the butylphthalide group, suggesting that butylphthalide combined with urinary kallidinogenase could effectively relieve the clinical symptoms of CCCI patients, and the effect was obvious. As one of the most important organs, the brain consumes 20% of the total oxygen consumption of the human body. It needs sufficient blood supply to support the normal activities. A decrease in cerebral blood flow affects the supply of oxygen to brain cells, leading to changes in brain function. CCCI is not focal cerebral ischemia, in which the blood supply to the brain is only slowly reduced rather than completely blocked. With the increase of patients' age, the tolerance of brain tissue to ischemia and hypoxia is significantly reduced, and the autoregulation function of cerebral blood vessels is significantly weakened. Even slight changes in cerebral blood supply will greatly affect cerebral blood flow and trigger CCCI (Wu, 2017). A previous study found that the abnormal cerebral blood flow in CCCI patients is as high as 97.96% (Liu et al., 2017). Therefore, active and effective measures should be given to improve the blood flow velocity, which is of great clinical significance. Cheng et al. (2016) pointed out that butylphthalide can significantly improve cerebral blood flow velocity in CCCI patients. Pan et al. (2020) revealed that butylphthalide injection has a definite therapeutic effect on acute cerebral infarction, which effectively alleviating the symptoms of neurological loss and improving cerebral blood flow in ischemic area. The results of this study showed that after treatment, the levels of MCA, VA and BA in the combined group were higher than those in the butylphthalide group, suggesting that butylphthalide combined with urinary kallidinogenase could effectively improve the cerebral blood flow velocity and blood circulation in patients with CCCI, thus improving the prognosis of patients.

CCCI is a chronic and progressive development process, and the cerebral perfusion of patients is in a state of long‐term hypoperfusion, which cannot meet the needs of normal brain metabolism, but has not reached the state of cerebral infarction. Therefore, attention should be paid to the cerebral blood flow perfusion status of patients in treating CCCI patients (Lv et al., 2013). Yang et al. (2021) found that Fasudil combined with urinary kallidinogenase has better clinical efficacy in the treatment of acute cerebral infarction patients, and has advantages in restoring neurological function and cerebral blood perfusion. Gao and Li (2021) pointed out that butylphthalide soft capsule can improve the level of cerebral blood flow perfusion in ischemic area of patients with symptomatic chronic internal carotid artery occlusion, inhibit the production of MPP‐9, and reduce inflammation. The results of this study showed that after treatment, the levels of rCBF and rCBV in the combined group were higher than those in the butylphthalide group, and the level of rMTT was lower than that in the control group, suggesting that butylphthalide combined with urinary kallidinogenase could effectively improve the cerebral blood circulation and alleviate blood hypoperfusion in patients with CCCI. This study further confirmed that there was no significant difference in the incidence of adverse events between the two groups, suggesting that the combination therapy would not increase the adverse events with high safety.

The study has some limitations. First, this is a retrospective study, selection bias is unavoidable. Second, the sample size is relatively small, a multicenter randomized controlled study should be conducted to further confirm the results of this study. Third, the evaluation of efficacy in this study is subjective to some extent, which may have a certain impact on the results of the study.

## 6 CONCLUSION

Butylphthalide combined with urinary kallidinogenase in the treatment of CCCI can accelerate blood flow speed, improve cerebral blood circulation, and alleviate blood hypoperfusion. This method has favorable clinical efficacy and no significant side effects, providing a new insight for the treatment of CCCI.

## CONFLICT OF INTEREST STATEMENT

The authors declare that they have no competing interests.

### PEER REVIEW

The peer review history for this article is available at https://publons.com/publon/10.1002/brb3.2920.

## Data Availability

The data that support the findings of this study are available from the corresponding author upon reasonable request.
